# Effects of Robotic-Assisted Gait Training in Children and Adolescents with Cerebral Palsy: A Network Meta-Analysis

**DOI:** 10.3390/jcm10214908

**Published:** 2021-10-24

**Authors:** Raquel Olmos-Gómez, Antonia Gómez-Conesa, Inmaculada Calvo-Muñoz, José A. López-López

**Affiliations:** 1 International Doctoral School of the University of Murcia (EIDUM), University of Murcia, 30100 Murcia, Spain; 2 Research Group Research Methods and Evaluation in Social Sciences, Mare Nostrum Campus of International Excellence, University of Murcia, 30100 Murcia, Spain; agomez@um.es; 3 Department of Physiotherapy, Faculty of Health Sciences, Catholic University San Antonio of Murcia, 30100 Murcia, Spain; inmaculada.calvo@um.es; 4 Department of Basic Psychology and Methodology, Faculty of Psychology, University of Murcia, 30100 Murcia, Spain; josealopezlopez@um.es

**Keywords:** cerebral palsy, robotic, gait, children, adolescents, network meta-analysis

## Abstract

Gait disturbances are common in children and adolescents with cerebral palsy (CP). Robotic-assisted gait training (RAGT) is becoming increasingly widespread, and hence it is important to examine its effectiveness. A network meta-analysis (NMA) of clinical trials comparing treatments with RAGT vs. other physical therapy treatments was carried out. This study was conducted according to the NMA version of the Preferred Reporting Items for Systematic Reviews and Meta-Analyses (PRISMA-NMA) guidelines and following the recommendations of the Cochrane Handbook for Systematic Reviews of Interventions. The outcome variables used were the D and E dimensions of the Gross Motor Function Measure (GMFM), gait speed, resistance, and stride length. Among 120 records, 8 trials were included. This NMA did not find statistically significant results for any of the comparisons examined in any of the outcomes studied and the magnitude of the effect size estimates was low or very low. Our NMA results should be interpreted with caution due to the high clinical heterogeneity of the studies included.

## 1. Introduction

Cerebral palsy (CP) refers to a group of movement and posture disorders caused by malformations or brain damage during early development, which limit activities of daily life and self-care. CP motor disorders are often accompanied by secondary musculoskeletal problems [1]. CP is the most common cause of physical disability in childhood, with a prevalence of 1.7 per 1000 live births in Europe [2]. Likewise, the early diagnosis of CP is important to help ensure specific opportunities for early care, aimed at optimizing future outcomes [3]. The prognosis of gait is mainly determined by the acquisition of certain gross motor patterns and the age at which they are reached, which are essential determinant of the development of independent walking [4].

An early and focused intervention strategy is recommended for CP and should be the standard of care to optimize neuroplasticity, prevent complications, and improve the functional abilities, participation, and quality of life of the child, as well as the well-being of the caregiver [5,6]. Children and adolescents with CP usually present alterations in body movement patterns, with impaired gait, which negatively affect their health and their ability to interact with their peers [7]. Therefore, a key therapeutic goal in children and adolescents with CP is to improve walking ability. Several treatment options are available from physiotherapy, including walking on the ground and on a treadmill with partial weight bearing [8].

There is evidence that children with CP whose treatment emphasizes functional activities have better clinical outcomes than those receiving movement-focused treatments [9]. Results also improve as the child’s motivation rises and an increase in the number and intensity of programmed exercises is achieved [10]. Scientific evidence suggests that functional therapies, characterized by movements significantly similar to motor skills, are effective in improving motor function in children with CP [9,10]. Additionally, robotic technologies have been adapted to a functional recovery of gait, so that robotic-assisted gait training (RAGT) allows a longer duration of training, at more variable speeds, and with a constant gait pattern tailored to the patient. This training, based on intensity and repetition of movement, has beneficial effects in the recovery and improvement of postural and locomotor functions in patients with neurological injury [11,12,13]. The robotic devices allow more intensive training, allowing to walk up to 1000 steps in a 20 min session, as opposed to the reeducation of walking on the ground with manual help—which reaches up to approximately 100 steps—and the treadmill, which achieves approximately 300–400 steps, limited by fatigue [11].

A meta-analysis published in 2017 [14] that included 10 studies, 2 of which were randomized controlled trials (RCTs), showed evidence that RAGT treatments produce benefits in people with CP aged 4–22 years, but did not establish a clear relationship between RAGT and gait improvements. That same year [15], a systematic review on gait impairment in different pediatric pathologies, which included 17 studies with different methodological designs of which only 2 could be meta-analyzed, determined weak and inconsistent evidence of the benefits of RAGT for gait disorders in children. However, none of these focused on children and adolescents with CP, nor did they take into account the configuration parameters of the device, nor compared the effects of different types of devices or therapy provided in order to determine the optimal type of treatment.

To resolve this issue, a network meta-analysis (NMA) allows multiple treatments to be simultaneously compared in a single analysis by combining direct and indirect evidence within a network of interventions compared across a set of studies [16].

The purpose of our study was to address this gap in knowledge by conducting a systematic review and a meta-analysis to synthesize the most recent evidence with higher quality designs, and to evaluate the comparative effectiveness of the different treatments. Furthermore, we performed NMA to examine whether robotic gait training systems are effective in improving gross motor function (related to standing and gait), and the characteristics of gait (speed, endurance, and stride length), in children and adolescents with CP.

## 2. Materials and Methods

A systematic review and NMA was carried out following the NMA version of the Preferred Reporting Items for Systematic Reviews and Meta-Analyses (PRISMA-NMA) recommendations [17]. The protocol for this meta-analysis was registered in the PROSPERO registry with code CRD42020176247.

### 2.1. Selection Criteria

To be included in our meta-analysis, studies had to meet the following criteria: (a) randomized controlled trials (RCTs) or controlled clinical trials (CCTs) as study designs; (b) samples of children and adolescents with CP (less than 18 years old); (c) treated with robotic gait training devices; (d) studies published up to March 2020; (e) written in English, Spanish, or French; (f) studies had to provide the necessary statistical data to calculate effect sizes; and (g) the sample had to include at least 5 subjects in each study group at the end of the intervention period(this threshold is frequently used in systematic reviews and meta-analyses on treatment effectiveness, in order to prevent making very imprecise effect estimates and to reduce the risk of confounding).

### 2.2. Information Sources

Combined study search strategies were implemented: (a) PubMed databases were consulted through the National Center for Biotechnology Information platform, the ILACS database and IBECS through the Virtual Health Library platform, PEDro, the Central database through the Cochrane Library platform, the Academic Search Complete database, CINAHL complete and Psycinfo through the Elton B. Stephens Co (EBSCO) platform, and the Web of Science and Scielo databases through the Web of Science platform; (b) the references of the articles found in the aforementioned databases were reviewed and a backward search of these references was carried out; (c) specialized journals were consulted and experts were contacted to locate published and unpublished studies.

### 2.3. Search Strategy

A search was carried out with the combination of the following keywords (descriptors): “cerebral palsy”; “robotic assisted gait training”; “robotic-assisted locomotor training”; “robotic-assisted therapy”; “lokomat”; “walkbot”; “robotic assisted treadmill”. All these terms were combined with the Boolean operators AND and OR. The search was carried out between the months of January and March 2020 (Appendix A).

### 2.4. Study Selection and Data Extraction

The selection of the studies was carried out by two investigators (R.O.G. and I.C.M.) independently and in two phases: duplicates and articles not meeting the selection criteria based on title and abstract were removed; and full-text articles of all remaining studies were then screened for inclusion. Disagreements regarding inclusion were resolved by discussion and consensus.

After identifying the studies, the moderator and outcome variables were defined, and two investigators (R.O.G. and I.C.M.) independently extracted the data from the included studies using an ad hoc data extraction form. In the event of disagreement, three investigators (R.O.G., I.C.M., and A.G.C.) rechecked the original article and followed with a discussion to reach a consensus.

To meet the objectives, data were extracted from included studies following the PICO strategy [18]. The moderating variables were classified into context variables: (a) place, (b) country; participants: (a) age of the subjects, (b) sex, (c) type of CP, (d) other pathologies, (e) functional level according to the Gross Motor Function Classification System (GMFCS-ER) [19,20], (f) use of assistive devices for walking, (g) use of lower limb orthoses, (h) comorbidity; treatment performed: (a) type of walking robot, (b) duration of treatment, (c) intensity of treatment, (d) magnitude of treatment, (e) use of virtual reality games, (f) if treatment with robot combined with physiotherapy is performed, (g) if they receive other different therapies, (h) if an established number of sessions was established, (i) the treatment parameters, (j) dimension of the International Classification of Functioning, Disability and Health (ICF) [21], (k) homogeneity of treatment, (l) follow-up, (m) informed consent; methodological variables: (a) study design, (b) method of assigning subjects to groups, (c) type of control group (active or inactive), (d) follow up, (e) sample size in the pre-test, post-test, and follow up, (f) post-test and follow-up mortality, (g) risk of bias (RoB) (using the Cochrane’s tool for assessing risk of bias scale) [22]; extrinsic variables: (a) date of publication, (b) professional training of the first author.

In order to assess the reliability of the coding process, Cohen’s kappa was calculated for qualitative variables and the intraclass correlation coefficient for quantitative variables [23].

### 2.5. Assessment of Risk of Bias

Cochrane’s tool for assessing risk of bias [22] was used to assess RoB, which establishes six levels of bias: selection biases, performance biases, detection biases, attrition biases, reporting biases, and other biases. Each item should be individually assessed in clinical trials, indicating a high or low level of bias or unclear risk of bias. In the event of disagreement, rechecking of the original article followed by discussion among the three co-authors (R.O.G., I.C.M. and A.G.C.) was used to reach a consensus.

### 2.6. Data Synthesis and Analysis

Outcome variables used were the D and E dimensions of the Gross Motor Function Measure (GMFM) scale of gross motor function [24], as well as the gait speed, the resistance measured with the 6 min walk test (6 mWT) [25], and the step length.

Prior to the integration of results, intervention effects were quantified at the arm level with the standardized mean change index, Cohen’s d, which was used as the effect size index. For its calculation, the equation of Becker (1988), Morris (2000), and Morris and DeShon (2002) [26] was used:

$${d_{c}}_{2}=\left[1-\frac{3}{4\left(n-1\right)-1}\right]\frac{\bar{\gamma }\;pre-\bar{\gamma }\;pos\;}{S\;pre}$$

Network plots were constructed to map the evidence available for each outcome, with the node size and line thickness proportional to the number of patients contributing to each intervention and intervention comparison, respectively.

An NMA was performed within a frequentist framework assuming a fixed-effects model. NMA is a technique for simultaneously comparing multiple treatments in a single analysis by combining direct and indirect evidence within a network of randomized controlled trials [16]. For each outcome, we conducted NMAs to pool all evidence in the network, and examined consistency using a generalized version of the Q statistic [27].

## 3. Results

### 3.1. Study Selection

**Figure 1:**
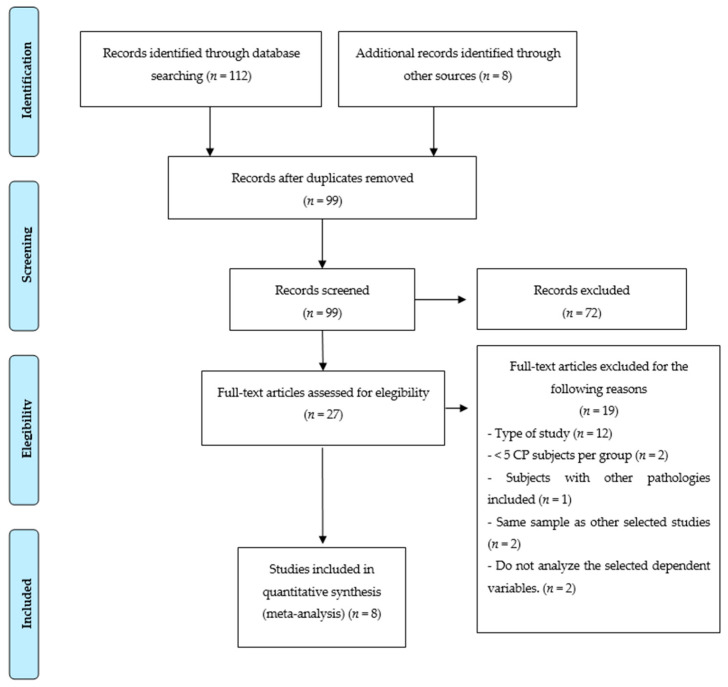
PRISMA flowchart of assessed studies.

The number of records identified through electronic searches was 112—in addition, 8 more articles were identified through other sources. After eliminating duplicate citations and screening, 27 full-text articles were evaluated for possible eligibility, of which 8 articles were finally included in the NMA [28,29,30,31,32,33,34,35] (Figure 1).

### 3.2. Trial Characteristics

Of the eight meta-analyzed studies, two were conducted in Italy [28,31], two in Turkey [34,35], one in Mexico [29], one in Poland [30], one in the USA [32], and one in France [33]. Three studies were carried out in hospitals [29,32,34], and the rest in ambulatory health centers and universities. The total number of subjects included in the studies was 217, with a mean age of 9.57 years, and an average 54.45% of male patients. Regarding the type of CP, five studies reported subjects with only bilateral spastic involvement [28,30,31,32,33], two studies with only unilateral spastic involvement [29,35], and one study included both conditions [34]. The GMFCS level ranged from I to IV, with level II being the most represented (included in all studies), followed by level III (five studies) [28,30,31,32,34]. Regarding the gait robot used, five studies used the Lokomat [29,30,31,33,34] and three studies used the 3DCaLT [32], Gait Trainer [28], and Innowalk-Pro [35], respectively. The number of RAGT sessions carried out varied from 10 to 40 sessions, with a duration of 30 to 45 min per session, which may or may not be accompanied by physiotherapy treatment. All participants in the control groups received physiotherapy treatment, the content of which varied across the different studies. Regarding the dimensions of the ICF [21], function was recorded in all studies, activity in five studies [28,31,32,34,35], and participation in one study [35]. In addition, all studies described a homogeneous treatment for each group, and except for one [30], the existence of informed consent was specified. With regard to follow up, six studies [28,29,31,32,34,35] carried one out, with times varying between 1 month [28] and 12 months [29]. Concerning methodological variables, all studies were clinical trials, although two of them were not randomized [31,35], and all had active control groups. The publication dates of the articles ranged from 2011 to 2019, and the authors were physiotherapists, doctors, or engineers. Table 1 and Table 2 summarize the characteristics of the subjects and the treatments. The reliability of the coding process yielded a Cohen *K* coefficient of 0.975(95% CI: 0.9652; 0.9848) and an intraclass correlation coefficient of 0.948 (range: 0.935–0.958).

### 3.3. Assessment of Risk of Bias

According to Cochrane’s tool for assessing risk of bias [22], we found a high RoB for selection due to the inappropriate generation of random sequences, in two studies [31,35]; for allocation concealment in one study [35]; for performance by blinding of participants and staff in two studies [32,35]; and for detection by the blinding of outcome assessment in two studies [32,35] (Appendix B).

### 3.4. Network Meta-Analysis

Table 3 shows the synthesis of the results of the outcome variables of the robot-treated groups and of the control groups of the studies.

**Figure 2:**
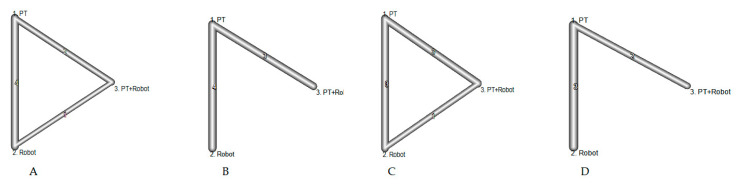
Comparisons of robot-assisted training vs. physiotherapy vs. physiotherapy + robot-assisted training: (**A**) gross motor function measure; (**B**) speed; (**C**) endurance; and (**D**) step length.

**Figure 3:**
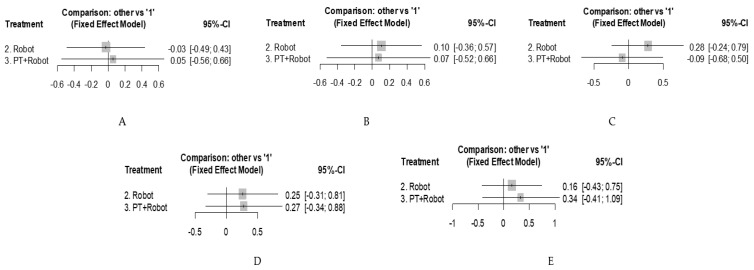
Forest plot of the standardized mean change index calculated for the comparisons of robot-assisted training vs. physiotherapy and physiotherapy + robot-assisted training vs. physiotherapy: (**A**) gross motor function measure dimension D; (**B**) gross motor function measure dimension E; (**C**) speed; (**D**) endurance; and (**E**) step length. ‘1’: physiotherapy.

Motor function, corresponding to dimensions D and E of the GMFM, was included in five studies [31,32,33,34,35]. We identified four comparisons between RAGT and physiotherapy treatment; two comparisons between combined RAGT treatment with physiotherapy and physiotherapy treatment; and one comparison between RAGT combined treatment with physiotherapy and treatment with RAGT (Figure 2A). For dimension D, there was no evidence of a difference between the combined treatment of RAGT with physiotherapy and physiotherapy alone (d = 0.05 95% CI: −0.56; 0.66)or RAGT alone and physiotherapy (d = −0.03, 95% CI: −0.49; 0.43) (Figure 3A). Regarding dimension E, results also suggested no difference when comparing isolated RAGT (d = 0.10, 95% CI: −0.36; 0.57) or combined treatment with physiotherapy (d = 0.07 (95% CI: −0.52; 0.66) vs. physiotherapy alone (Figure 3B).

Gait speed was included in seven studies [28,29,30,32,33,34,35]. We identified three comparisons between the combined treatment of RAGT with physical therapy and physiotherapy treatment, and four comparisons between RAGT and physiotherapy treatment (Figure 2B). The comparison between the RAGT treatment and physiotherapy obtained an effect size d = 0.28 (95% CI: −0.24; 0.79), while the combined treatment vs. physiotherapy showed an effect size d = −0.09 (95% CI: −0.68; 0.50) (Figure 3C). These results also suggested no difference between treatments.

Resistance was reported in five studies [28,31,32,34,35]. We identified three comparisons between the combined treatment of RAGT with physical therapy and physiotherapy treatment, three comparisons between RAGT treatment and physiotherapy treatment, and one comparison between RAGT combined treatment with physiotherapy and RAGT treatment (Figure 2C). Both the RAGT treatment vs. physiotherapy, with an effect size d = 0.25 (95% CI: −0.31, 0.81) and the combined RAGT treatment with physiotherapy vs. physiotherapy alone, with an effect size d = 0.27 (95% CI: −0.34, 0.88), did not show differences between them and physiotherapy treatment (Figure 3D).

Step length as measured in five studies [28,30,32,33,34]. We identified two comparisons between the combined treatment of RAGT with physical therapy and physiotherapy treatment, and three comparisons between RAGT and physiotherapy treatment (Figure 2D). The comparison between treatment with RAGT and physiotherapy, with effect size d = 0.16 (95% CI: −0.43; 0.75) and combined treatment of RAGT with physiotherapy vs. physiotherapy, with effect size d = 0.34 (95 % CI: −0.41; 1.09) showed no evidence of a difference (Figure 3E).

Following Cohen’s guidelines [36], the observed effect sizes can be considered as very low (d < 0.2), for all comparisons on gross motor function GMFM D and E, and gait speed; and low for the variables’ resistance and stride length (Table 4). Furthermore, between-design Q statistics suggested no evidence of inconsistency in any of the networks (Table 5).

Network plots were constructed to map the evidence available for each outcome, with the node size and line thickness proportional to the number of patients contributing to each intervention and intervention comparison, respectively.

## 4. Discussion

The aim of this meta-analysis was to examine whether treatments that use RAGT are effective in children and adolescents with CP to improve both gross motor function in standing and walking, as well as the characteristics of the gait with respect to speed, resistance, and step length. In gross motor function, dimensions D and E of the GMFM were included since they are related to the motor function of gait and standing.

To our knowledge, this is the first NMA comparing the effectiveness of RAGT with other treatment alternatives in children and adolescents with CP.

This NMA did not find statistically significant results for any of the comparisons examined in any of the outcomes studied and the magnitude of the effect size estimates was low or very low according to Cohen [36]. Nonetheless, the general trend of the results pointed towards the improvement of children and adolescents who used RAGT, both isolated and combined with physiotherapy, in all outcome variables. Specifically, the isolated RAGT treatment further improved the dimension of the GMFM (walking, running, and jumping) and gait speed; moreover, the combined treatment of RAGT with physiotherapy showed greater improvement in the D dimension of the GMFM (standing), in the resistance measured with 6 mWT and in the step length.

Regarding the types of robot used in RAGT, five of the clinical trials used the Lokomat [29,30,31,33,34], and the others used the Gait Trainer [28], the Innowalk Pro [35], and the 3DCaLT [32], respectively. There are some differences between robots. The Lokomat is a fixed exoskeleton that is used over a treadmill, the Gait Trainer and the Innowalk Pro are distal effect robots, and the 3DCaLT is a custom designed 3D cable-driven robotic gait training system. There are also differences between the physiotherapy treatments performed in the clinical trials included in this NMA. The type of physiotherapy intervention performed in every clinical trial is described in Table 2. Due to the low number of studies and the wide range of ages and GMFCS-ER levels across the included studies, it is not possible to make specific recommendations based on the results of our study. Nonetheless, this synthesis provides insights on the state of the art in this context and hence it may guide future primary studies.

Our NMA included eight clinical trials (two non-randomized), published in the last decade (2011–2019). Previous meta-analyses included 2 RCTs (out of 10 studies) [14], or only 2 studies [15]. Despite the fact that the overall trend in the results suggested an increase in gait speed and endurance and an improvement in gross motor function in dimensions D and E of the GMFM, when robotic devices were used in children and adolescents with CP classified in levels I and II of the GMFCS-ER, the effect estimates provided no evidence to back such claims at this point (gait speed d = 0.21 [−0.09; 0.51], endurance d = 0.21 [−0.06; 0.49], D dimension of GMFM d = 0.18 [−0.10; 0.45], and E dimension d = 0.12 [−0.15; 0.40]) [14]. Similarly, no differences in gait speed were found between RAGT and exercise or another physiotherapy treatment [15].

A review of RAGT for people with CP concluded that its use promotes physical and cognitive integration, and such a combination is expected to lead to better treatment outcomes [37]. Regarding selective voluntary motor control in children with CP, a recent review identified the use of RAGT in three studies, one of them in lower extremities, and the results did not show evidence that RAGT was superior to a program delivered at home [38].

Other published articles have investigated the effectiveness of RAGT in children with CP, but they were not included in this NMA because they did not meet the inclusion criteria. Some of these show evidence of a general improvement, mainly in gait speed and endurance [13,39,40,41,42], and in gross motor function (dimensions D and E of the GMFM) [39,40,41,42,43,44,45], and therefore show some promise for RAGT to be considered as a therapeutic option in the pediatric setting.

### 4.1. Limitations

There are several limitations to this study, mainly the integration of a small number of clinically heterogeneous studies due to the restrictive selection criteria, some of them with a high or unclear risk of bias. Furthermore, the scarce evidence available prevented us from differentiating the effectiveness of RAGT by age ranges or by GMFCS-ER levels.

The difference among studies in terms of robot types, robot parameters, dose and training time used, as well as physiotherapy treatments performed, also limits the recommendations on these aspects in the use of RAGT.

### 4.2. Implications for Practice and Research

RAGT is a treatment alternative to improve gross motor function in gait motor function, gait speed and endurance, and step length in children and adolescents with CP, and when combined with physiotherapy treatments, it also improves gross motor function of standing; however, no differences were found compared to physiotherapy treatments. Our NMA allowed us to explore a greater range of comparisons between interventions.

With regard to future clinical studies, researchers in this field should aim to strengthen the evidence within network arms of each treatment by recruiting larger samples and adopting a more standardized framework to designing, implementing, and reporting interventions. Likewise, researchers should focus on the effect of the treatments in the medium and long term, with follow ups that enable the examination of effectiveness over time.

## 5. Conclusions

Although there is evidence to suggest that RAGT treatments are effective in children and adolescents with CP, our NMA of clinical trials found no significant differences among RAGT, RAGT combined with physical therapy, and physical therapy treatments to improve the outcomes we examined; thus, we cannot make a clinical recommendation regarding which of these treatment options should be preferred.

Limitations of the current evidence include a high risk of bias and high clinical heterogeneity. We recommend large higher-quality RCTs, including head-to-head comparisons of RAGT, as these would provide stronger scientific evidence in this field.

## Figures and Tables

**Table 1:** Characteristics of patients for included studies.

Study	N		Country	Mean Age		% Male		CP Type		GMFCS Level	
	EG	CG		EG	CG	EG	CG	EG	CG	EG	CG
Smania et al., 2011 [28]	9	9	Italy	13.88	12.79	44.44	66.67	Spastic bilateral		I, II, IV	I, III, IV
Arellano-Martínez et al., 2013 [29]	8	6	Mexico	7.5	6.83	25	83.33	Spastic unilateral		II	
Druzbicki et al., 2013 [30]	26	9	Poland	10.1	11	54	54	Spastic bilateral		II, III	II
Peri et al., 2017 [31]	12/10/12 ^1^	10	Italy	8/6.8/10.8 ^1^	9.3	50/40/58.33 ^1^	50	Spastic bilateral		I, II, III	
Wu et al., 2017 [32]	11	12	USA	11.3	10.5	54.55	66.67	Spastic bilateral		I, II, III, IV	
Wallard et al., 2018 [33]	14	16	France	8.3	9.6	57.14	43.75	Spastic bilateral		II	
Aras et al., 2019 [34]	10	10/9 ^2^	Turkey	9.3	9.3/9.3 ^2^	60	60/66.7 ^2^	Spastic bilateral, Spastic unilateral		II, III	
Yazici et al., 2019 [35]	12	12	Turkey	8	9	50	50	Spastic unilateral		I, II	

N: sample; EG: experimental group; CG: control group; CP: cerebral palsy; GMFCS: Gross Motor Function Classification System. ^1^ The data correspond to experimental group 1: intervention with robot; experimental group 2: intervention with robot + physiotherapy 10 weeks; experimental group 3: intervention with robot + physiotherapy 4 weeks. ^2^ data correspond to control group 1: partial body weight support treadmill exercise. (PBWSTE)/control group 2: antigravity treadmill exercise (ATE).

**Table 2:** Characteristics of treatments for included studies.

Study	N		Robot	Number of Sessions		Session Time (min)		EG Includes Physiotherapy	CG Includes Physiotherapy	Type of Physiotherapy Intervention
	EG	CG		EG	CG	EG	CG			
Smania et al., 2011 [28]	9	9	GaitTrainer	10	10	30 + 10	40	Yes	Yes	Stretching, joint mobilizations, strength exercises, balance, and gait exercises
Arellano-Martínez et al., 2013 [29]	8	6	Lokomat	10	10	30	30	No	Yes	Gait in hydrotherapy tank
Druzbicki et al., 2013 [30]	26	9	Lokomat	20	20	45	-	Yes	Yes	Motor control, increasing stability in the sitting and upright positions, developing walking skills
Peri et al., 2017 [31]	12/10/12 ^1^	10	Lokomat	40/20 + 20/20 + 20 ^1^	40	30/30/30 ^1^	-	No/Yes/Yes ^1^	Yes	Gait training, balance, functional skills, strength, stretching
Wu et al., 2017 [32]	11	12	3DcaLT	18	18	30–40	30–40	No	Yes	Gait treadmill training
Wallard et al., 2018 [33]	14	16	Lokomat	20	20	40	-	No	Yes	Unspecified physiotherapy
Aras et al., 2019 [34]	10	10/9 ^2^	Lokomat	20	20/20 ^2^	45	45/45 ^2^	No	Yes	- PBWSTE - ATE
Yazici et al., 2019 [35]	12	12	Innowalk-Pro	36	-	30	-	Yes	Yes	Active functional strength exercises, stretching, squats, stair climbing, functional reach, balance board, single leg balance

N: sample; EG: experimental group; CG: control group; Min: minutes; ^1^ data correspond to experimental group 1: intervention with robot; experimental group 2: intervention with robot + physiotherapy 10 weeks; experimental group 3: intervention with robot + physiotherapy 4 weeks. ^2^ data correspond to control group 1: partial body weight support treadmill exercise (PBWSTE)/control group 2: antigravity treadmill exercise. (ATE).

**Table 3:** Results of the outcome variables of the experimental groups (robot interventions) and control groups. (d pre-test-post-test (95%CI)).

Study	GMFM-D		GMFM-E		Speed		Endurance		Step Length	
	EG	CG	EG	CG	EG	CG	EG	CG	EG	CG
Smania et al., 2011 [28]					0.268 (−0.557; 1.093)	−0.108 (−0.88; 0.664)	0.506 (−0.46; 1.472)	0.022 (−0.74; 0.784)	0.602 (−0.439; 1.643)	−0.602 (−1.643; 0.439)
Arellano-Martínez et al., 2013 [29]					−0.055 (−0.884; 0.774)	−0.178 (−1.246; 0.89)				
Druzbicki et al., 2013 [30]					0.139 (−0.275; 0.553)	0.258 (−0.561; 1.077)			0.121 (−0.291; 0.533)	0.09 (−0.678; 0.858)
Peri et al., 2017 [31]	0.069 (−0.568; 0.706)/0.168 (−0.565; 0.901)/ 0.136 (−0.511; 0.783) ^1^	0.11 (−0.611; 0.831)	0.075 (−0.562; 0.712)/0.04 (−0.671; 0.751)/0 (−0.633; 0.633) ^1^	0.062 (−0.651; 0.775)			0.095 (−0.544; 0.734)/0.081 (−0.634; 0.796)/−0.092 (−0.731; 0.547) ^1^	0.014 (−0.697; 0.725)		
Wu et al., 2017 [32]	0.05 (−0.62; 0.72)	0.332 (−0.379; 1.043)	0.057 (−0.613; 0.727)	0.061 (−0.574; 0.696)	0.14 (−0.544; 0.824)	−0.057 (−0.692; 0.578)	0.529 (−0.333; 1.391)	−0.017 (−0.65; 0.616)	0.369 (−0.401; 1.139)	0.186 (−0.473; 0.845)
Wallard et al., 2018 [33]	0.393 (−0.281; 1.067)	0.125 (−0.416; 0.666)	0.555 (−0.205; 1.315)	0.08 (−0.455; 0.615)	0.664 (−0.163; 1.491)	0.095 (−0.442; 0.632)			0.627 (−0.177; 1.431)	0 (−0.531; 0.531)
Aras et al., 2019 [34]	0.191 (−0.55; 0.932)	0.259 (−0.505; 1.023)/0.422 (−0.487; 1.331) ^2^	0.13 (−0.595; 0.855)	0.171 (−0.564; 0.906)/0.389 (−0.499; 1.277) ^2^	0.305 (−0.481; 1.091)	0.305 (−0.481; 1.091)/0 (−0.762; 0.762) ^2^	0.273 (−0.497; 1.043)	0.227 (−0.526; 0.98)/0.38 (−0.504; 1.264) ^2^	0 (−0.71; 0.71)	0 (−0.71; 0.71)/0.903 (−0.404; 2.21) ^2^
Yazici et al., 2019 [35]	0.344 (−0.373; 1.061)	0.281 (−0.409; 0.971)	0.303 (−0.397; 1.003)	0.122 (−0.521; 0.765)	−0.563 (−1.402; 0.276)	−0.1 (−0.741; 0.541)	1.243 (−0.129; 2.615)	0.375 (−0.356; 1.106)		

GMFM-D: gross motor function measure dimension D; GMFM-E: gross motor function measure dimension E; EG: experimental group; CG: control group. ^1^ data correspond to experimental group 1: intervention with robot; experimental group 2: intervention with robot + physiotherapy 10 weeks; experimental group 3: intervention with robot + physiotherapy 4 weeks; ^2^ data correspond to control group 1: partial body weight support treadmill exercise (PBWSTE)/control group 2: antigravity treadmill exercise (ATE).

**Table 4:** Results for components.

OUTCOME	PT d (CI)	ROBOT d (CI)	t^2^	I^2^
GMFM D	0.082 [−0.573; 0.738]	0.054 [−0.557; 0.664]	0	0% [0.0%; 0.0%]
GMFM E	−0.033 [−0.677; 0.610]	0.071 [−0.523; 0.665]	0	0% [0.0%; 20.2%]
Speed	−0.368 [−1.153; 0.417]	−0.091 [−0.682; 0.499]	0	0% [0.0%; 21.3%]
Endurance	0.020 [−0.659; 0.700]	0.273 [−0.336; 0.883]	0	0% [0.0%; 50.7%]
Step Length	0.174 [−0.778; 1.127]	0.336 [−0.414; 1.086]	0.094	23.8% [0.0%; 88.3%]

GMFM D: gross motor function measure dimension D; GMFM E: gross motor function measure dimension E; PT: physiotherapy; t^2^: between-study variance estimate; I^2^: inconsistency; d: mean effect size; CI: 95% confidence interval.

**Table 5:** Between-design Q statistics.

Outcome	Q_BD_	df	*p*
GMFM D	0.00	2	0.999
GMFM E	0.12	2	0.943
Speed	0	0	-
Endurance	0.95	2	0.622
Step Length	0	0	-

GMFM D: gross motor function measure dimension D; GMFM E: gross motor function measure dimension E; Q_BD_: between-design Q statistic; df: degrees of freedom; *p*: *p*-value. Outcomes with df = 0 had no potential for inconsistency (e.g., only direct or indirect evidence was available for each treatment comparison).

## Appendix A

**Table A1:** Data sources and searches.

Data Sources	Search Strategy	Records Identified	Selected Studies
PubMed	((((((“cerebral palsy”) AND “robotic assisted gait training”) OR “robotic-assisted locomotor training”) OR “robotic-assisted therapy”) OR “lokomat”) OR “walkbot”) OR “robotic assisted treadmill”	37	16
PEDro	(1) Abstract and title: robotic-assisted Topic: cerebral palsy Method: clinical trial (2) Abstract and title: Robotic training Topic: cerebral palsy Method: clinical trial	8	1
Web of Science	“cerebral palsy” AND (“robotic assisted gait training” OR “robotic-assisted locomotor training” OR “robotic-assisted therapy” OR “lokomat” OR “walkbot” OR “robotic assisted treadmill”)	36	1
Cochrane	“cerebral palsy” AND (“robotic assisted gait training” OR “robotic-assisted locomotor training” OR “robotic-assisted therapy” OR “lokomat” OR “walkbot” OR “robotic assisted treadmill”)	22	0
Psycinfo	“cerebral palsy” AND (“robotic assisted gait training” OR “robotic-assisted locomotor training” OR “robotic-assisted therapy” OR “lokomat” OR “walkbot” OR “robotic assisted treadmill”)	9	1
Ibecs	“cerebral palsy” AND (“robotic assisted gait training” OR “lokomat” OR “walkbot” OR “robotic assisted treadmill”)	0	0
Lilacs	“cerebral palsy” AND (“robotic assisted gait training” OR “lokomat” OR “walkbot” OR “robotic assisted treadmill”)	0	0

## Appendix B

**Table A2:** Risk of bias: Cochrane’s collaboration tool for assessing risk of bias.

Study	Sequence	Allocation	Blinding1	Blinding2	Outcome1	Outcome2	Other
Smania et al., 2011 [28]	+	+	?	?	+	+	+
Arellano-Martínez et al., 2013 [29]	+	?	?	?	?	?	+
Druzbicki et al., 2013 [30]	+	?	?	+	+	?	+
Peri et al., 2017 [31]	-	?	?	?	+	+	+
Wu et al., 2017 [32]	+	+	-	-	+	+	+
Wallard et al., 2018 [33]	+	+	?	?	+	?	+
Aras et al., 2019 [34]	+	+	?	?	+	+	+
Yazici et al., 2019 [35]	-	-	-	-	?	+	+

Sequence: random sequence generation; Allocation: allocation concealment; Blinding1: blinding of participants and personnel; Blinding2: blinding of outcome assessment; Outcome1: incomplete outcome data; Outcome2: selective reporting. Low risk (+); high risk (-); unclear risk (?).
